# Plant Polyamines

**DOI:** 10.3390/plants9040511

**Published:** 2020-04-16

**Authors:** Taku Takahashi

**Affiliations:** Graduate School of Natural Science and Technology, Okayama University, Okayama 700-8530, Japan; perfect@cc.okayama-u.ac.jp; Tel.: +81-86-251-7858

**Keywords:** copper amine oxidase, polyamine oxidase, spermidine, spermine, stress response, thermospermine

## Abstract

Polyamines are small organic compounds found in all living organisms. According to the high degree of positive charge at physiological pH, they interact with negatively charged macromolecules, such as DNA, RNA, and proteins, and modulate their activities. In plants, polyamines, some of which are presented as a conjugated form with cinnamic acids and proteins, are involved in a variety of physiological processes. In recent years, the study of plant polyamines, such as their biosynthetic and catabolic pathways and the roles they play in cellular processes, has flourished, becoming an exciting field of research. There is accumulating evidence that polyamine oxidation, the main catabolic pathway of polyamines, may have a potential role as a source of hydrogen peroxide. The papers in this Special Issue highlight new discoveries and research in the field of plant polyamine biology. The information will help to stimulate further research and make readers aware of the link between their own work and topics related to polyamines.

Polyamines are polycationic compounds ubiquitously found in all organisms and play various roles by interacting with negatively charged macromolecules, such as DNA, RNA, and proteins. They are also present as a conjugated form with organic molecules and proteins. In plants, polyamines have been shown to regulate growth, development, and responses to biotic and abiotic stresses. In recent years, our understanding of plant polyamines in terms of their biosynthesis, metabolism, and physiological function has seen major advances [1,2,3]. In this situation, I decided to edit a Special Issue aimed at highlighting new discoveries and advances in plant polyamine research.

One unique feature of plant polyamines is the widespread occurrence of thermospermine from primitive algae to land plants [4]. However, it was only after the turn of the century that the specific function of thermospermine in regulating xylem development of vascular plants was discovered. The gene for thermospermine synthase had initially been identified as the gene for spermine synthase from a mutant of Arabidopsis, which shows an excess xylem development with a dwarf growth phenotype, but it was later proven to code for thermospermine synthase [5]. In accordance with its vascular-specific expression, *AtPAO5*, a gene encoding polyamine oxidase (PAO) that predominantly catalyzes thermospermine, also shows vascular-specific expression in Arabidopsis [6,7,8]. Sagor et al. [9] report on their finding that the level of thermospermine and the hypersensitivity to thermospermine in the Arabidopsis *pao5* mutant is restored by transgenic expression of its homologue *SelPAO5* from *Selaginella lepidophylla*, a primitive vascular plant related to ferns. Interestingly, SelPAO5 converts thermospermine to norspermidine instead of spermidine. On the other hand, our study focused on the effect of exogenous thermospermine on vasculature in a monocot plant and found that it has a repressive effect on xylem development in rice crown roots [10]. The study also revealed that the genes encoding apoplastic PAOs are drastically activated by both spermine and thermospermine.

Unlike other plant growth regulators, which generally control the expression of genes via activation of specific transcription factors, polyamines have been shown to act in mRNA translation. A paper by Poidevin et al. [11] clearly and concisely reviews the involvement of spermidine and thermospermine in regulating mRNAs at the level of translation. The authors discuss the significance of these polyamines in the context of mRNA quality control.

There is accumulating evidence that polyamine oxidation, the main catabolic pathway of polyamines, may have a potential role as a source of hydrogen peroxide (H_2_O_2_). A review by Yu et al. [12] provides recent information on plant PAOs, especially those in three model species: rice, Arabidopsis, and tomato. Toumi et al. [13] addressed a potential link between polyamine catabolism and heat-shock proteins, HSP90s, under heat stress using Arabidopsis mutants in combination with pharmacological experiments and revealed that heat stress-induced increase in acetylated spermidine and spermine is further enhanced in plants having reduced levels of HSP90s. Because the increased polyamine titers under heat stress were associated with the increase in PAO-derived H_2_O_2_, the authors suggest an antagonistic relationship between HSP90s and polyamines/PAOs/H_2_O_2_ homeostasis.

The Arabidopsis genome contains two genes for spermidine synthase, one gene for spermine synthase, one gene for thermospermine synthase, and five genes for PAOs, that exhibit different or overlapping substrate specificity [10]. By contrast, there are ten putative genes encoding copper amine oxidase (CuAO) annotated in the Arabidopsis genome [14]. This might reflect the functional divergence of this enzyme. CuAOs catalyze the intracellular and extracellular terminal catabolism of amines, including monoamines, diamines and polyamines [15]. The expression of these genes is inducible by stress-related hormones such as methyl-jasmonate, abscisic acid (ABA), and salicylic acid, or by wounding, and they have been shown to function in wound healing, defense against pathogens, protoxylem differentiation, and stomatal closure [16]. However, not all genes have been characterized in terms of the physiological roles so far. A study by Fraudentali et al. [17] addresses the function of AtCuAOδ and provides data showing that AtCuAOδ is involved in the H_2_O_2_ production related to ABA-induced stomatal closure. Recently, the authors also revealed that leaf-wounding induces expression of another CuAO gene, *AtCuAOβ*, in root vascular tissues [18].

Polyamines are present in cells at relatively high concentrations in the mM range, suggesting their potential importance as a source of nitrogen. A classical study showed that putrescine and spermidine could be a substitute for inorganic nitrogen for the growth of explants from the Jerusalem artichoke tubers [19]. The interplay among polyamines and nitrogen could be a key issue in the plant growth. A review by Paschalidis et al. [20] focuses on how polyamines and nitrogen regulate plant growth, especially under stress conditions. The authors also put focus on the possibility of crop improvement by genetically altering polyamine homeostasis. A paper by Anwar et al. [21] provides evidence that tomato fruit architecture can be altered by the transgenic expression of yeast spermidine synthase gene. Previous studies by these authors have shown that the increased polyamine content in transgenic tomato fruits enhances nutritional quality, juice quality, and shelf life [22,23]. The obovoid phenotype observed in tomato fruits with higher content of polyamines is shown to be associated with increased expression of fruit shape-related and cell division genes [21].

A study by Labarrere et al. [24] gives insight into a different aspect of polyamine research. The authors investigated two metabolite classes, amines and quercetins, belonging to the flavonoid family, in three *Ranunculus* species native to Kerguelen Islands. The data suggest functional redundancy and versatility of these metabolites within species, which may be attributable to adaptation to unknown past environments or neutral microevolutionary differentiation among populations.

In summary, this Special Issue contains six research articles and three reviews. I believe that these papers will further stimulate research in this exciting filed of plant biology and make readers aware of the link between their own work and topics related to polyamines.
